# A Case of Multi-focal Osteonecrosis in the Context of Liver Transplant Following Ingestion of Amanita phalloides Mushroom Toxin

**DOI:** 10.7759/cureus.19513

**Published:** 2021-11-12

**Authors:** Peggy E Miller, Paula McQuail, Charlotte F Doran, Kevin McSorley, Paul Curtin

**Affiliations:** 1 Trauma and Orthopaedics, St. Vincent's University Hospital, Dublin, IRL; 2 Orthopedics, St James's Hospital, Dublin, IRL

**Keywords:** avascular osteonecrosis, knee avascular necrosis, amanita phalloides, death cap mushroom, liver transplant, multifocal osteonecrosis, acute total hip replacement

## Abstract

We present the case of a 44-year-old female who presented with atraumatic avascular necrosis (AVN) of the patella and hips bilaterally, following ingestion of the deadly fungus *Amanita phalloides* or ‘death cap’ and subsequent liver transplant. Upon presentation, in the hours following ingestion, our patient required a liver transplant and ICU admission. She was treated by a multidisciplinary team, with input from various specialities. Our patient required steroids in the months following this event. Six months after the liver transplant and subsequent ICU admission, our patient developed hip pain, thus limiting her mobility, ability to engage in physiotherapy and rehabilitation. X-rays were performed that excluded any acute pathology. She was still receiving high-dose steroids at this time. When the pain did not resolve with analgesia, MRI of pelvis and knee was performed and the patient was found to have polyarticular AVN. Acute bilateral total hip replacement was performed and within weeks, the patient returned to physiotherapy and to full rehabilitation. Conservative management of the patella was favoured. Over two years later, the patient can now mobilise independently.

The role of acute total hip replacement is evident in this case, and how in performing this surgery, the overall conditioning and health of our patient improved drastically. Currently, cases reporting *A. phalloides* ingestion are few and we wish to use this case to highlight the differential diagnosis in a patient presenting with joint pain in this context of fungus ingestion, organ transplant or prolonged steroid use.

## Introduction

*Amanita phalloides* or ‘death cap’ is one of the most dangerous variants of fungi found in Western Europe and North America. While hepatic and renal necrosis are widely reported following ingestion of this toxin, multifocal osteonecrosis is yet to be described. We present the simultaneous polyarticular functional decline and challenging management in a patient following such an occurrence in the context of a liver transplant and prolonged course of steroids. Additionally, we specifically document and illustrate the rare occurrence of atraumatic patellar osteonecrosis as a component of her presentation.

A 44-year-old female presented to our institution with fulminant hepatic failure and subsequent multi-organ failure following ingestion of *A. phalloides* mushrooms. She became bed-bound due to intractable lower limb pain after liver transplant and diffuse dermal necrosis requiring multiple skin grafts. Plain film and MRI investigations revealed bilateral Ficat stage 4 avascular necrosis (AVN) of both hips with associated florid myositis. The overlying skin on her left hip had ongoing serous ooze from the split thickness skin graft sites, exacerbating limited mobility. She underwent synchronous bilateral Corail Pinnacle (DePuy Synthes, Warsaw, IN) acute total hip replacements. Further radiological investigation revealed right patellar osteonecrosis.

## Case presentation

Patient XY, a 44-year-old female presented to the ED with vomiting following consumption of locally foraged mushrooms. An experienced forager, the patient had collected and prepared the mushrooms for dinner. Between six to eight hours later, the patient noted abdominal pain and associated vomiting. The vomitus was bilious in nature with multiple episodes occurring over the next two to three hours. With persistent vomiting, she called an ambulance that brought her to her local hospital. Admission and investigation found a deranged liver profile, and the diagnosis of acute liver failure secondary to the fungi consumption was made. Ms. XY was transferred to the National Liver Unit where she underwent orthotopic liver transplant. Following a tumultuous post-operative course including an ICU admission, and Hepatic, Renal, Plastics, Rheumatology and Dermatology input, an incidental finding on CT of the abdomen/pelvis foreshadowed the orthopaedic trajectory of our patient.

Following discharge from the ICU, the patient began recovery on the ward. At this point, her primary concern was painful discolouration at the pulps of her fingers and toes. Painful lesions were also noted in the right flank region. Dermatology input resulted in a diagnosis of skin necrosis post-transplant with microvascular thrombosis of the hands and feet. Skin graft to the right flank region was performed by the Plastics team. The post-operative period was once again complicated by sepsis and treated with antibiotics. She remained on a prolonged course of steroids for up to six months post-operatively.

**Figure 1:**
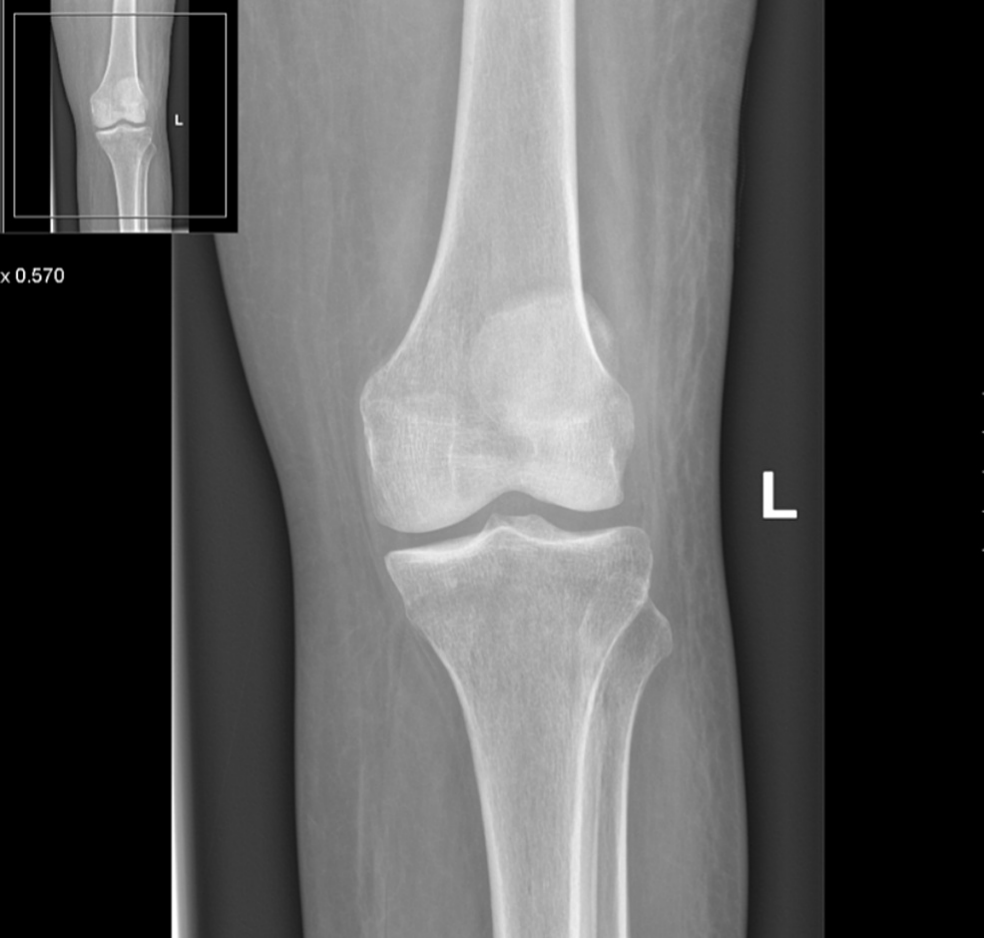
Anteroposterior view of the left knee excluding any acute fracture

**Figure 2:**
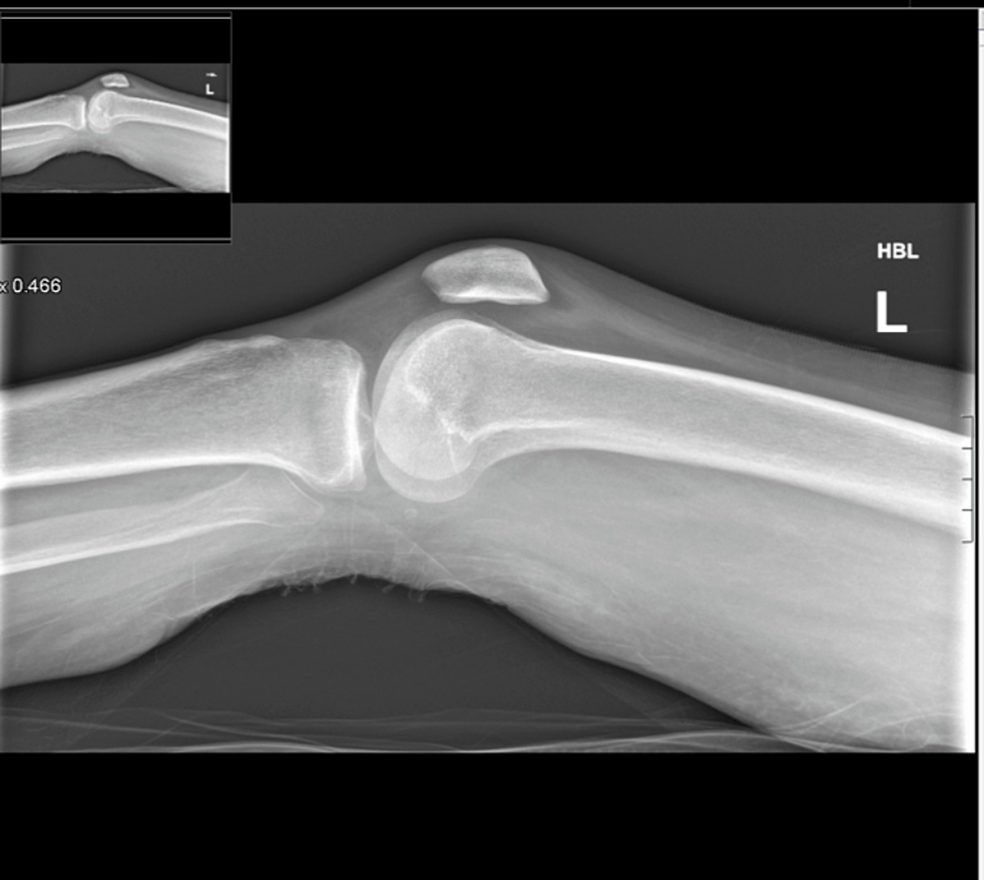
Lateral view of the left knee excluding any acute fracture

As XY continued to improve clinically, her recovery was aided by our multi-disciplinary team input. Throughout this period, the patient noted a new onset of groin pain resulting in regression in her mobility. In the proceeding four weeks, Ms. XY continued to suffer from pain, impeding her progress with physiotherapy. A physical exam now revealed a swollen and erythematous knee, with a reduced range of motion. An X-ray excluded knee pathology including joint effusion and fracture and confirmed normal alignment, as shown in Figure 1 and Figure 2. The Pain team now became involved and recommended increased medication doses and frequency.

**Figure 3:**
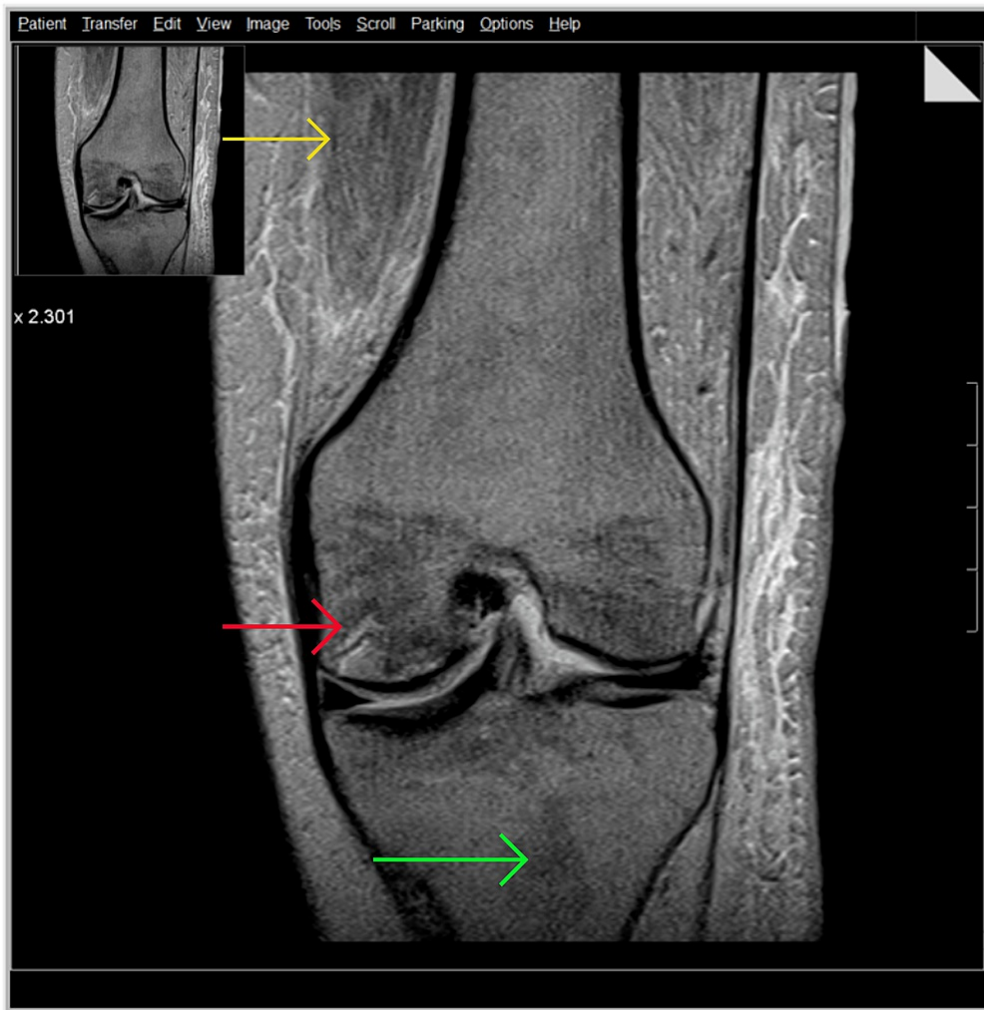
Knee MRI coronal view Yellow arrow identifies atrophic musculature; red arrow highlights areas of alternating hyper and hypointensity and serpiginous lines within the subchondral marrow of the medial and lateral femoral condyles; green arrow shows bone marrow heterogeneity.

**Figure 4:**
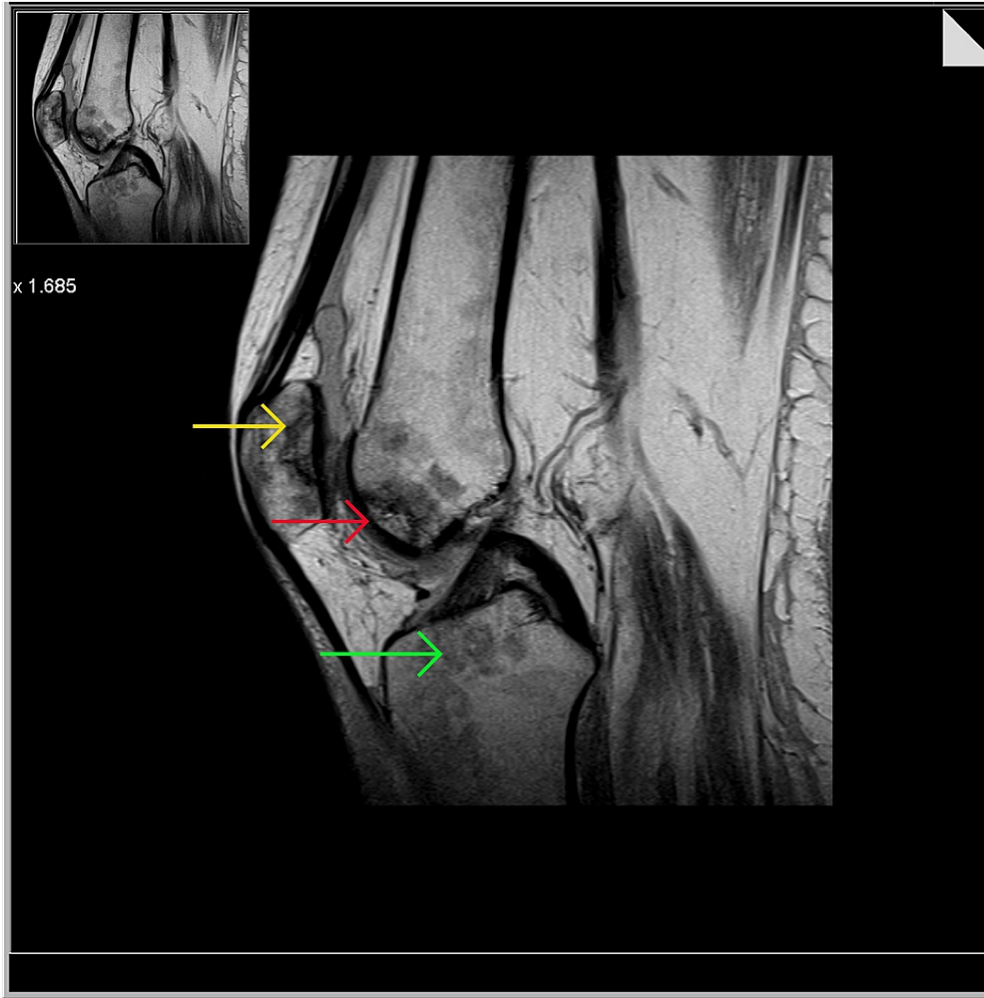
Knee MRI sagittal view showing avascular necrosis of the patella Yellow arrow is showing serpingous lines in the patella; red arrow is showing serpingous lines; green arrow is showing bone marrow heterogeneity

XY’s pain continued to increase over the subsequent six weeks and she was unable to engage fully in physiotherapy for six months. XY described the pain as sharp, without radiation and aggravated by movement. A physical exam revealed an oedematous swollen joint surrounding the cruciate ligament. An MRI was conducted as suggested by the pain team to investigate the integrity of the surrounding soft tissues. The cruciate ligaments, the menisci and both the quadriceips and patellar tendons were found to be intact. The associated musculature was atrophic. Several serpiginous lines within the subchondral marrow of the medial and lateral femoral condyles and in the patella with alternating hyper- and hypointensity were noted. Heterogenous surrounding marrow was also visualized, all of which was depicting AVN and a reactive sclerosis. Orthopaedic intervention was not advised. Evidence is provided in Figure 3 and Figure 4.

**Figure 5:**
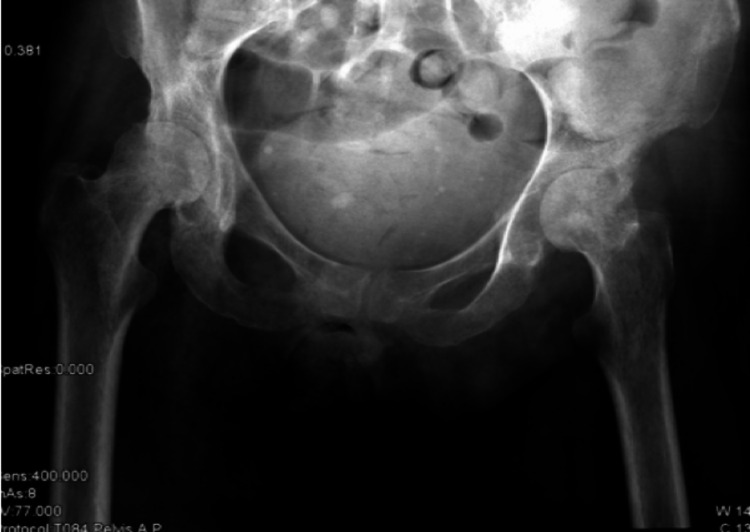
Anteroposterior view of the pelvis showing nil acute pathology

**Figure 6:**
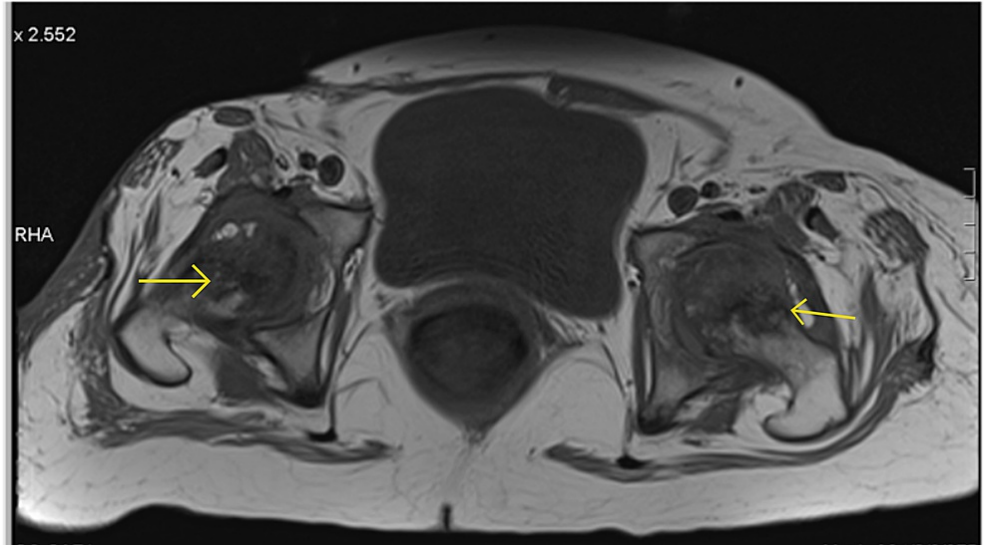
Pelvis MRI axial view Yellow arrows show bilateral sepiginous low-signal circumscribed areas in both femoral heads.

**Figure 7:**
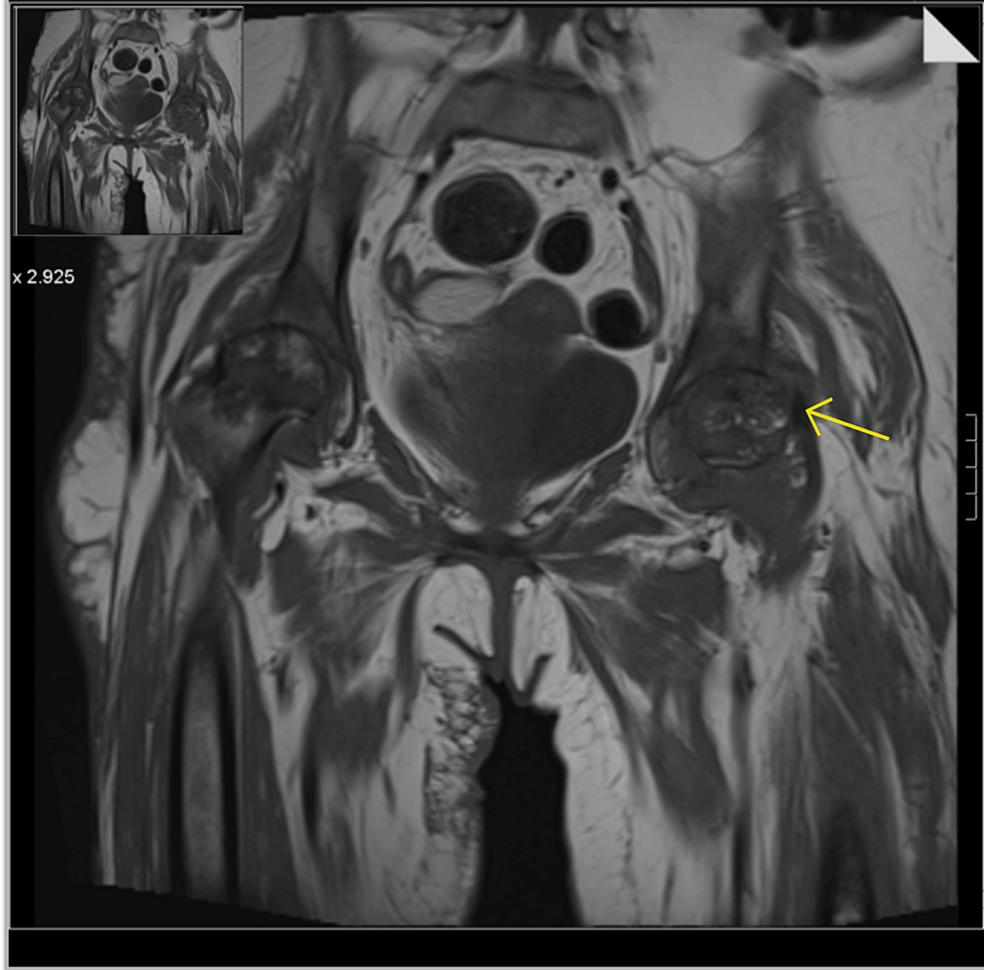
Pelvis MRI coronal view Yellow arrow shows the subchondral collapse in the superolateral aspect of the left femoral head

XY was in bed almost 24 hours a day and was experiencing bilateral hip pain. Normal X-Ray is shown in Figure 5. However, pelvis MRI now revealed marked bilateral femoral head oedema, with bilateral sepiginous low-signal circumscribed areas also noted in both femoral heads, with advanced loss of joint space and articular cartilage bilaterally (Figure 6). A subchondral collapse in the superolateral aspect of the left femoral head was noted (Figure 7). Complex effusions were described bilaterally, thus showing a secondary synovial osteochondromatosis. Muscular oedema was noted throughout; the right rectus muscle was severely atrophic. Avascular necrosis was confirmed.

Orthopaedic input

**Figure 8:**
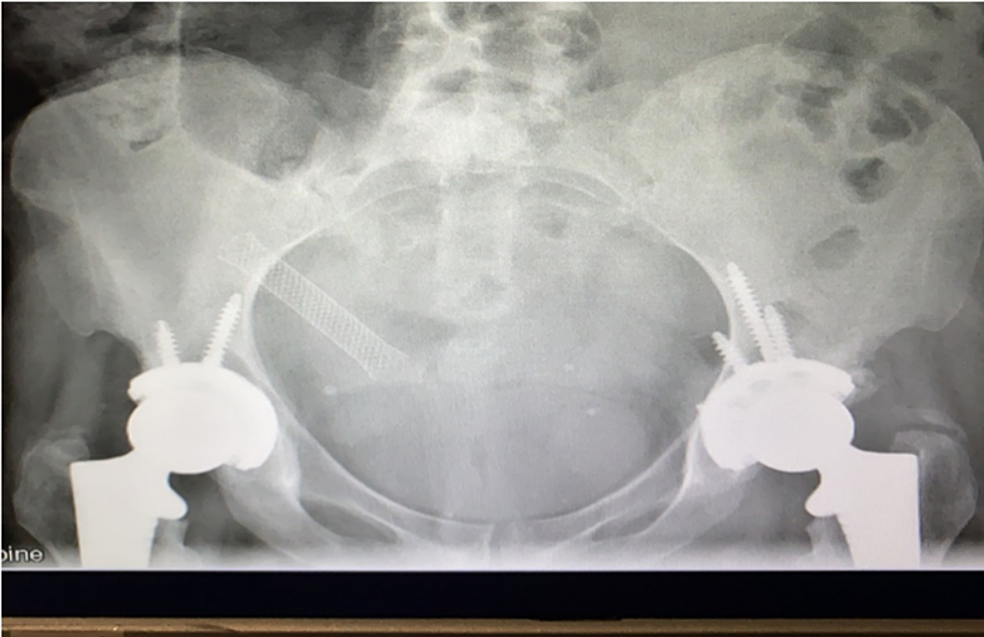
Pelvis X-ray with bilateral prostheses in situ

One week later, the patient underwent bilateral total hip replacements. Left femoral neck osteotomy was performed from the posterior approach and Multihole Pinnacle size 50 was inserted. A 48-mm cup was inserted on the right side from the posterior approach. The surgery was conducted under a general anaesthetic and then a spinal anaesthetic. Jubilee dressings were applied bilaterally, and continued for up to three weeks post-operatively. Serous ooze was noted particularly on the left side. She was treated with a course of intra-venous antibiotics and the ooze resolved. All clips had been removed by day 21 post-operatively and there were no complications noted. The patient restarted her physiotherapy program, using a wheelchair. Figure 8 shows the bilateral prostheses in situ.

The patient was mobilising up to 20 metres with the assistance of a frame, denied any pain and was increasing muscle strength and mobility daily. Objectives for discharge included the patient being able to mobilise independently at home and use a wheelchair outside. The patient was satisfied with this result and continued with physiotherapy and rehabilitation.

Over 18 months later, the patient is mobilizing independently at home and using crutches while outside. Despite complications post-transplant, she maintains good mobility and range of movement in her lower limbs.

## Discussion

Avascular necrosis following ingestion of *A. phalloides* has not been described previously in the literature. The specific cause of AVN in this case is unclear, although AVN following liver transplant is a well-known complication. The prolonged course of steroids taken by our patient also acted as a risk factor. Overall, AVN of the patella remains a rare phenomenon.

*A. phalloides* is a medium-sized mushroom with a white-domed cap. It is found in woodland areas most commonly in the summer/autumn seasons. While described as fatal in foraging guides, caution is recommended as other species of *Amanita *are considered edible (*A. fulva*) or *Agaricus*, only the gills of which differentiate the species.

A study carried out in the Mayo Clinic examined predictive factors for fractures and AVN in those undergoing liver transplant for primary sclerosing cholangitis and primary billiary cirrhosis, and cited risks such as pre-transplant fractures, osteopenia rates and glucocorticoid use post-transplant. Bone bloods for our patient were completed post-operatively showing no abnormalities. Our patient remained on steroid treatment for a prolonged course post-transplant that may have acted as a risk factor in her case [1]. AVN may also be described in patients with rheumatological associations post-transplant. Another case study in a 31-year-old male with AVN following transplant came to a diagnosis of anti-phospholipid syndrome, when three years post-operatively, the lupus anti-coagulant was identified and when anti-coagulant therapy commenced, the episodes of AVN stopped [2].

The link between transplant surgeries and AVN was first highlighted in 1975 in a study describing 29 cases in renal transplant patients. It noted a rate of 18% post-operatively and highlighted sites of AVN that were often unusual and bilaterally affected. The rapid evolution of AVN in the lower limb in radiological studies is also described, in keeping with the trajectory of our patient [3]. A more recent study in 2019 following 805 patients post-renal transplant found an incidence level of 4% for AVN. Risk factors identified included increased BMI and higher cumulative corticosteroid doses. It also found weight-bearing joint sites most frequently affected, such as hips and knees, as in our patient. This study recommends radiological screening in patients deemed high risk using MRI or bone scintigraphy [4].

Despite the associations between transplant and AVN, it may not be the sole cause in this case. Rare causes of AVN have been identified such as in the femoral head in the case of haemolytic uraemic syndrome (HUS) in a 19-year-old male following an outbreak of Shiga-toxin-producing *Escherichia coli* in a US army base. It was summarised by the authors that intravascular dissemination caused by HUS may have caused micro-occlusion in the vessels of the femoral head [5]. Disseminated intravascular coagulation is a common complication post-liver transplant. It is perhaps this similar coagulation environment that allows AVN to develop.

AVN following toxin ingestion is a known phenomenon. A recent study discussed AVN in patients following paraquat ingestion, a commonly used herbicide in the Eastern world, with a rate of up to 18% [6].

During her post-operative course, our patient was admitted to the ICU following systemic inflammatory response syndrome, the precise cause of which is unknown. This prolonged inflammatory state may have caused ischaemic insult to the blood supply of the femoral heads, also affecting the patella. This has been described previously in a patient following multi-organ failure and subsequent joint pain a few months later [7].

Non-traumatic cases of AVN are caused by vascular compromise, bone and cell death and defective bone repair [8].

The efficacy of the surgical intervention is undisputed here. Surgery is not the first-line treatment in patients with AVN. Medical treatments such as nonsteroidal anti-inflammatory drugs (NSAIDs) and bisphosphonates, or prevention with anti-coagulation and statins may be recommended. However, medical treatments were limited in this case as NSAIDs and statins are contra-indicated in liver transplant recipients. Joint replacement provided a definitive and simple approach avoiding complications and interactions within a multi-morbid patient [9,10]. The role of an acute total hip replacement in an in-patient population is also highlighted in this case. This intervention allowed for systemic improvement and an increase in general conditioning in our patient’s overall health. Without this intervention, it is likely that her respiratory effort would have remained compromised, skin quality further deteriorated and mood symptoms associated with chronic pain, slow progress and reduced mobility aggravated. The acute bilateral total hip replacement allowed our patient to engage fully with physiotherapy and regain mobility and independence following an almost fatal accident.

While avascular necrosis or osteonecrosis may present multi-focally, osteonecrosis of the patella remains relatively rare. Osteonecrosis of the knee joint is categorized by cause: spontaneous, secondary or post-arthroscopic [11]. In a retrospective study conducted at the Johns Hopkins University School of Medicine, of all patients presenting with osteonecrosis of the knee, only 9% received a diagnosis of avascular necrosis of the patella. Only one patient in the study experienced pain on the anterior aspect of the joint, and the most common cause of avascular necrosis of the patella was deemed to be corticosteroid use. This was also thought to be proportionate to the steroid dose, and many patients presenting with AVN of the knee also suffered from Crohn’s or chronic obstructive pulmonary disease (COPD), and frequently would have been prescribed steroids in the past. The superior pole of the patella was the only part of the patella affected in patients in the study. It is uncommon for the proximal tibia or fibula to be concomitantly affected with the patella. No surgical intervention was performed for patients in the study, although long-term sequelae of the disease included total knee arthroplasty requirement [12]. The difficulty surrounding the diagnosis of patellar AVN is also highlighted as radiological features may be present on only 20% of plain film X-rays, arguing the necessity of MRI or bone scintigraphy in making the diagnosis [13]. A previous study examining 120 knees of 60 patients diagnosed with avascular necrosis of the femoral head found 10 patients to also have osteonecrosis of the patella, a distribution pattern in keeping with that of our case [14].

The most significant issues that need answers are as follows: Should we be screening all patients with prolonged corticosteroid use for AVN using MRI? Should services treating patients with an increased BMI, such as diabetic screening or bariatric services, be used for the condition? Is an orthopaedic review ample or are radiological investigations warranted, despite the risks associated with radiation? Should this be a priority in an already overburdened public system such as that in the Republic of Ireland where patients may wait up to two years for an out-patient MRI?

## Conclusions

While AVN remains an uncommon condition, several risk factors have been identified including mushroom toxin ingestion, liver transplant and prolonged steroid use amongst others. This case report describes a patient with an unusual trajectory and varying risk factors for the condition. We hope that through this case report, clinicians may consider AVN as a possible cause of new-onset joint pain in patients with complex medical backgrounds.
